# Will we reach the Sustainable Development Goals target for tuberculosis in the European Union/European Economic Area by 2030?

**DOI:** 10.2807/1560-7917.ES.2019.24.12.1900153

**Published:** 2019-03-21

**Authors:** Hanna Merk, Csaba Ködmön, Marieke J van der Werf

**Affiliations:** 1 European Centre for Disease Prevention and Control, Stockholm, Sweden

**Keywords:** tuberculosis, TB, sustainable development, public health surveillance, registries

## Abstract

We assessed progress towards the Sustainable Development Goals target for tuberculosis in the European Union/European Economic Area using the latest tuberculosis (TB) surveillance and Eurostat data. Both the TB notification rate and the number of TB deaths were decreasing before 2015 and the TB notification rate further declined between 2015 and 2017. With the current average decline in notification rate and number of TB deaths however, the EU/EEA will not reach the targets by 2030.

In 2015, all United Nations Member States adopted the 17 Sustainable Development Goals (SDGs) and their 169 targets [1]. The target for tuberculosis (TB) is to end the epidemic by 2030. The End TB Strategy provides three additional sub-targets that are used to measure progress towards the SDGs [2]. According to these sub-targets, the TB incidence should be 80% lower in 2030 compared with 2015; the number of TB deaths should be 90% lower and no family should face catastrophic costs due to TB. Here, we assess progress towards the first two sub-targets at European Union/European Economic Area (EU/EEA) level. Information on catastrophic costs is not available at EU/EEA level.

## Analysis

We used data obtained from the European tuberculosis surveillance network under the joint coordination of the European Centre for Disease Prevention and Control (ECDC) and World Health Organization (WHO) Regional Office for Europe [3] and data from Eurostat [4] for the years 2008–17. The TB case data were extracted from The European surveillance system (TESSy) [5] hosted by ECDC (as at 5 October 2018). Since Croatia did not report case-based TB data to TESSy for 2008–11, Croatia was excluded from the notification rate for those years. The population denominators for the notification rates were obtained from Eurostat (as at 20 April 2018), as were the data on cause of death due to TB (ICD 10 code A15-A19 and B90, as at 21 November 2018) [6]. Cause of death data were only available up to 2015 (last updated by EUROSTAT on 20 July 2018). The TB notification rates were used as proxy for TB incidence and reported TB deaths as a proxy for actual number of deaths due to TB.

Countries with missing annual data on deaths and reporting 10 or less deaths per year in the remaining years were considered to have zero TB deaths for the years with missing data. Denmark did not report any data on TB deaths in 2010 but reported more than 10 deaths in the other years. We therefore estimated the number of TB deaths, by calculating the average of the two preceding and following years and applying the ceiling function in STATA version 14.2 (StataCorp, College Station, Texas, United States).

To assess whether the EU/EEA will reach the SDG target we used the average annual change in notification rate between 2008 and 2017 and the average annual change in number of deaths between 2008 and 2015 and assumed that the change will continue similarly in future.

For our analysis, we used STATA/SE 14.2.

## Key findings

**Figure 1:**
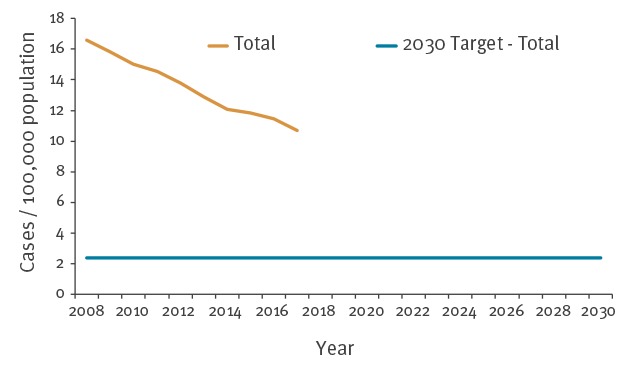
Tuberculosis notification rate over time and the End TB Strategy 2030 sub-target for Sustainable Development Goal 3, European Union/European Economic Area, 2008–2030

The total TB notification rate declined during the study period (Figure 1). In 2017, the notification rate was 10.7 per 100,000 population in the EU/EEA, resulting in an overall decline of 10% since 2015. The average annual decline between 2008 and 2017 was 4.8%.

Blue line: 80% reduction in tuberculosis notification rate compared with 2015.

An 80% reduction in TB notifications in the EU/EEA in 2030 compared with 2015 results in a target TB notification rate of 2.4 per 100,000 population.

If the 4.8% average annual decline continued unchanged, the EU/EEA would reach a TB notification rate of 5.7 per 100,000 population in 2030. The annual average decline required to reach the target is 10.9%.

**Figure 2:**
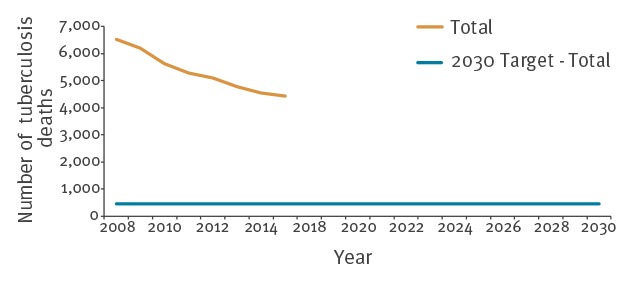
Number of deaths due to tuberculosis over time and the End TB Strategy 2030 sub-target for Sustainable Development Goal 3, European Union/European Economic Area, 2008–2030

The total number of TB deaths declined during the study period (Figure 2). In 2015, the number of registered TB deaths was 4,437. Progress since 2015 cannot be measured as there is no available data after 2015 at EU/EEA level. The average annual decline between 2008 and 2015 was 5.3%.

Blue line: 90% reduction in the number of tuberculosis deaths compared with 2015.

A 90% reduction in TB deaths in the EU/EEA in 2030 compared with 2015 results in a target of 444 TB deaths per year.

If the 5.3% average annual decline continued unchanged, the EU/EEA would reach 1,947 TB deaths per year in 2030. The annual average decline required to reach the target is 14.2%.

## Discussion

The targets for TB incidence and the number of TB deaths set in the End TB Strategy translate to 2.4 TB cases per 100,000 population and 444 TB deaths for the EU/EEA in 2030. Both the annual TB notification rate and the number of TB deaths were decreasing before 2015 and the TB notification rate further declined by 10% between 2015 and 2017. If the average 4.8% annual decline of the TB notification rate continues in the EU/EEA we will not reach the target by 2030; the average 5.3% decline of TB deaths is also not sufficient to reach the target.

Compared to other parts of the world, the observed TB notification rate and number of TB deaths in the EU/EEA are low [7]. Nonetheless, the SDG and End TB Strategy targets apply to the EU/EEA and our results show that there is little room for complacency.

Globally, the average annual decline of the TB incidence rate was 1.5% between 2000 and 2017, far less than what was observed in the EU/EEA [7]. The global number of TB deaths among HIV-negative people decreased by 5% since 2015 and by 29% between 2000 and 2017 [7]. Since 33% of the reported TB cases in the EU/EEA are diagnosed in individuals of foreign origin [8], a decrease in the global incidence of TB may impact the observed TB incidence in the EU/EEA. This would be more apparent in countries that diagnose a large proportion of their TB cases in individuals of foreign origin such as Malta, Norway and Sweden (> 85% of TB cases of foreign origin).

Within the EU/EEA, some countries are closer to ending TB than others: 24 countries reported a notification rate of less than 10 TB cases per 100,000 population [8]. In addition, there are substantial differences in the annual change in the TB notification rate in the EU/EEA [8]. In the period 2013–17, two countries observed an increasing notification rate of more than 5% per year, 17 had a decreasing notification rate of more than 5% and in 11, the annual change was between - 5% and + 5%. To reach the target of 2.4 TB cases per 100,000 population, different strategies may need to be applied within the EU/EEA countries, depending on the respective epidemiological situation. Similarly, the estimated TB deaths among HIV-negative persons in EU/EEA countries in 2017 ranged between zero and 920, with an annual percentage change ranging from -17.6% to 15.0% between 2013 and 2017 [8]. Thus further indicating that some EU/EEA countries are making more progress towards the target than others are.

The End TB Strategy includes a package of interventions that are encouraged for use by countries to prevent and control TB and reach the targets [2]. The interventions are grouped under three pillars: (i) integrated, patient-centred care and prevention, (ii) bold policies and supportive systems, and (iii) intensified research and innovation. Countries are encouraged to adapt their strategy to the specifics of their TB epidemic. In 2017, 17 EU/EEA countries had a national TB control plan or strategy [9].

It is acknowledged that specific actions are needed in countries that are close to ending TB and aiming for TB elimination [10]. These countries will often have epidemics concentrated in hard-to-reach and vulnerable populations e.g. migrants, prisoners and homeless people. Targeting hard-to-reach and vulnerable populations requires specific interventions and may need additional resources [11-14]. For example, screening and providing treatment for latent TB infection (LTBI) prevents new TB cases [15,16], as well as screening and treating prisoners and migrants for active TB may also contribute to a further decline [12,13]. To our knowledge, however, not all EU/EEA countries test hard-to-reach populations for LTBI. Mathematical modelling and cost-effectiveness studies show that programmatic management of LTBI can have an impact on TB burden [17,18]. In addition, migrants are not screened for TB in all EU/EEA countries [19]. The interventions suggested in the ECDC guidance on TB control in vulnerable and hard to reach populations are also not implemented in all countries in the EU/EEA [11].

Our results come with limitations. We used notification rate as a proxy for TB incidence. We believe this to be a valid approach since several studies have shown that completeness of TB surveillance data in EU/EEA countries is > 80% [20-22]. We relied on the completeness and accuracy of the cause of death register for the number of TB deaths. The quality of death registration systems has been assessed as good in most countries of the WHO European Region [23]. We therefore consider our results valid for assessing progress towards the targets on an EU/EEA level. However, accurately assigning cause of death is challenging [24] and improvements in cause of death registration may still be needed on country level [25]. We recognise that improvements in both TB surveillance and cause of death registration can affect the progress assessment if more complete data become available.

In conclusion, additional interventions need to be implemented to reach the targets for TB incidence and number of TB deaths in the EU/EEA, and thus the SDG, especially in countries that are currently facing stable or increasing trends.
